# Supplementary figures and images for: Alterations in the nasopharyngeal microbiome associated with SARS-CoV-2 infection status and disease severity
Source: PLoS One. 2022 Oct 14;17(10):e0275815. doi: 10.1371/journal.pone.0275815 (PMC9565700; doi:10.1371/journal.pone.0275815)

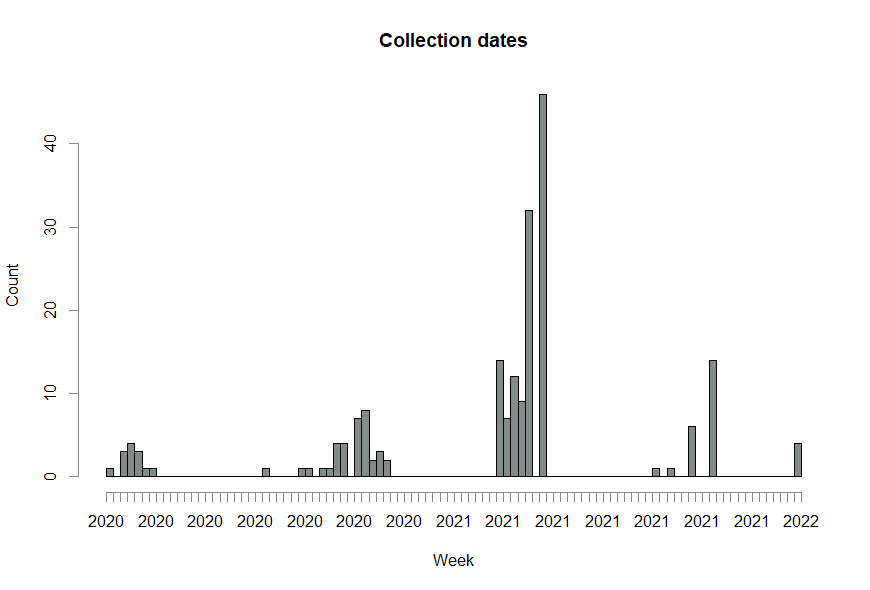

Supplement: S1 Fig — (PNG) [file pone.0275815.s001.png]

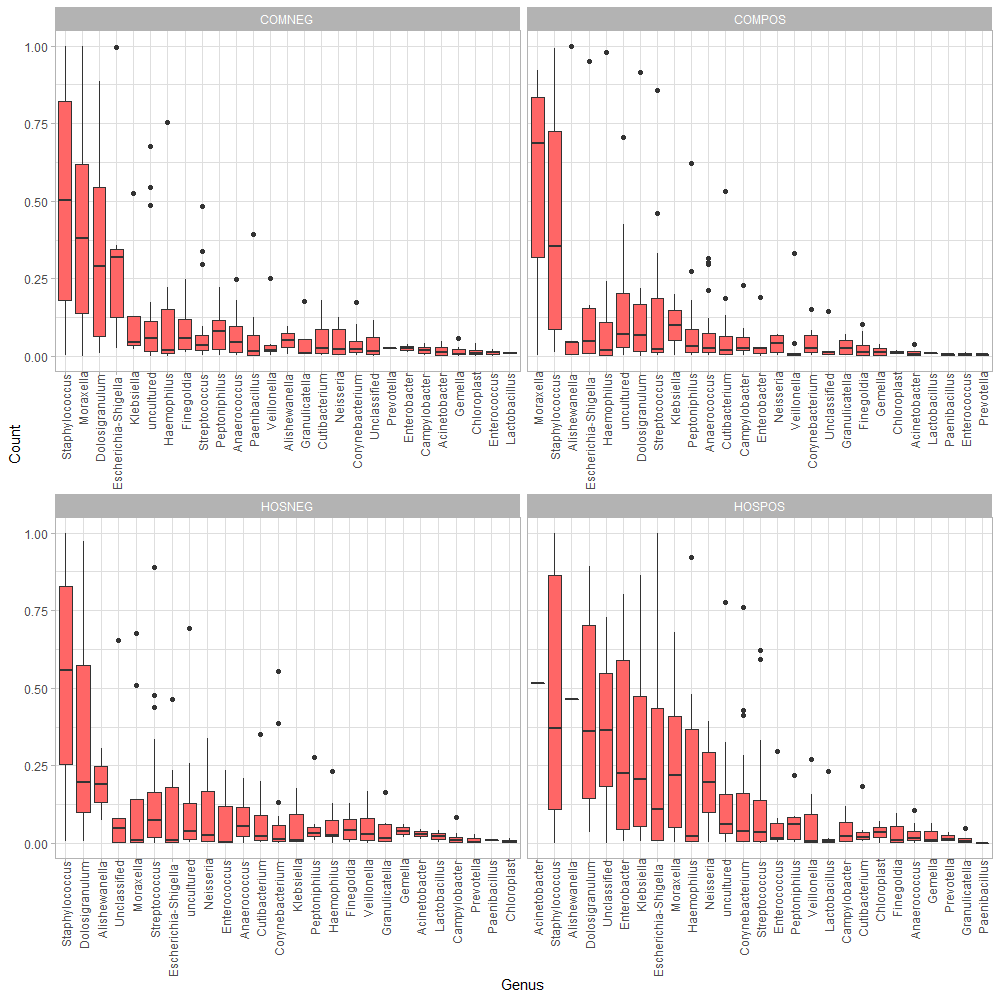

Supplement: S2 Fig — (PNG) [file pone.0275815.s002.png]

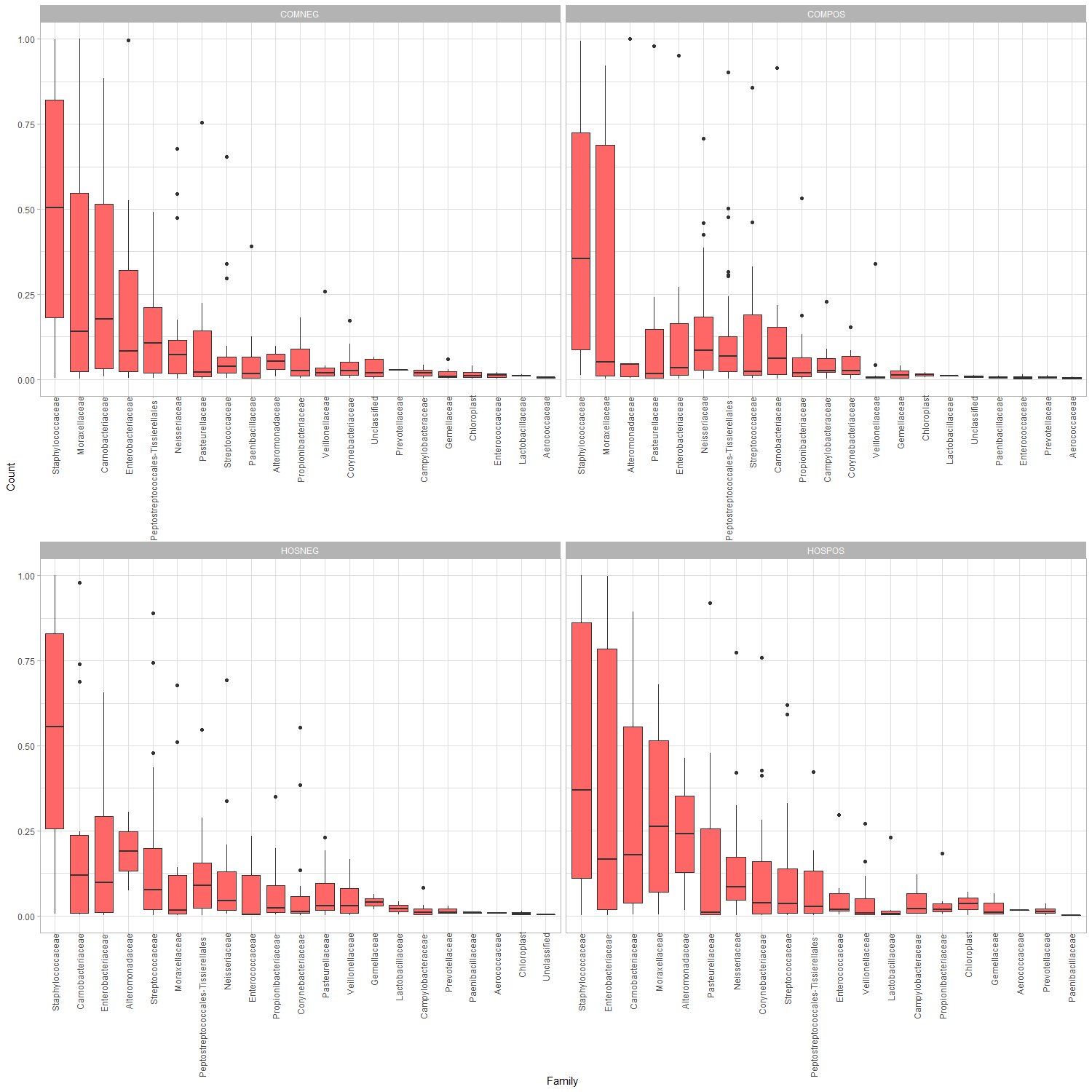

Supplement: S3 Fig — (PNG) [file pone.0275815.s003.png]
