# Supplementary material for: Alterations in the nasopharyngeal microbiome associated with SARS-CoV-2 infection status and disease severity
Source: PLoS One. 2022 Oct 14;17(10):e0275815. doi: 10.1371/journal.pone.0275815 (PMC9565700; doi:10.1371/journal.pone.0275815)
Supplement: S1 File — (DOCX) [file pone.0275815.s004.docx]

**Supplementary Tables/Figures**

**S1 Table. A. Primers, B. probes, and C. reaction conditions for Variant of Concern PCR testing**

| **A. Primers** | | | |
| --- | --- | --- | --- |
| **Primer name** | **target** | **Sequence 5’-3’** | **Design** |
| N501Y_E484K-F1 | E484K | AGAGAGATATTTCAACTGAAATCTATCAGG | Tracy Lee, BCCDC |
| N501Y-R3 | E484K & N501Y | CCACAAACAGTTGCTGGTGC | Tracy Lee, BCCDC |
| N501Y-F2 | N501Y | AATTGTTACTTTCCTTTACAATCATATGG | Tracy Lee, BCCDC |
| WT_N501Y_block | N501Y | CCAAC+C+CA+C+T+A+A/3InvdT/ | Tracy Lee, BCCDC |
| E_Sarbeco_F1 | E-gene | ACAGGTACGTTAATAGTTAATAGCGT | Corman et al. |
| E_Sarbeco_R2 | E gene | ATATTGCAGCAGTACGCACACA | Corman et al. |

| **B. Probes** | | | |
| --- | --- | --- | --- |
| **Target** | **Probe Dye** | **Sequence 5’-3’** | **Design** |
| E484K_MGB-P1 VIC | VIC-MGB | CTTGTAATGGTGTTAAAGGT | Tracy Lee |
| N501Y-P_FAM MGB | FAM-MGB | CCAACCCACTTATGG | Tracy Lee |
| E_Sarbeco_probe | CY5-TAO | ACACTAGCCATCCTTACTGCGCTTCG | Corman et al. |

**C. Thermocycling program**

| **Step** | **Cycles** | **Temperature** | **Time** |
| --- | --- | --- | --- |
| RT | 1 | 50^0^C | 5 minutes |
| Enzyme Activation | 1 | 95^0^C | 20 seconds |
| Amplification | 40 | 95^0^C | 3 seconds |
|  |  | 60^0^C | 30 seconds |


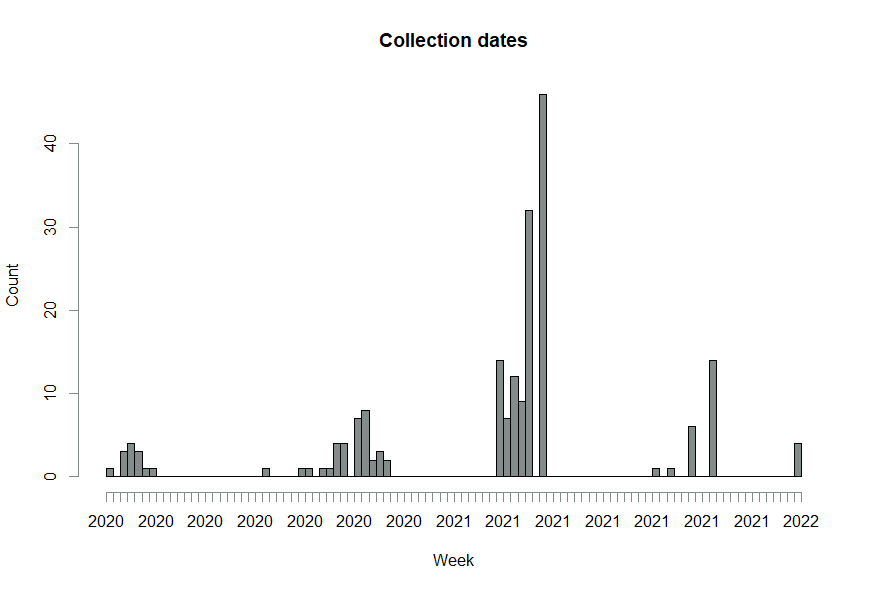


**S1 Fig. Histogram of collection dates for study specimens.** Data is stratified by week of the year and “count” represents the number of specimens at each week.

**S2 Table.** Distribution of RT-PCR C_t_ values among study specimens, including BioFire positives, which do not provide a C_t_ value.

| **Sample ID** | **Rdrp** | **E** | **ORF** | **BioFire** |
| --- | --- | --- | --- | --- |
| COMPOS1 |  |  | 16.9 |  |
| COMPOS10 |  |  | 18.4 |  |
| COMPOS11 |  |  | 28.1 |  |
| COMPOS12 |  |  | 19.8 |  |
| COMPOS13 |  |  | 23.2 |  |
| COMPOS14 |  |  | 23.4 |  |
| COMPOS15 |  |  | 35.7 |  |
| COMPOS16 |  |  | 22.5 |  |
| COMPOS17 |  |  | 19.2 |  |
| COMPOS18 |  |  | 18.1 |  |
| COMPOS19 |  |  | 18.2 |  |
| COMPOS2 |  |  | 15.8 |  |
| COMPOS20 |  |  | 28.5 |  |
| COMPOS21 | 21 | 20.9 |  |  |
| COMPOS22 |  |  | 19.6 |  |
| COMPOS23 |  |  | 23.5 |  |
| COMPOS24 |  |  | 18.3 |  |
| COMPOS25 |  |  | 31.8 |  |
| COMPOS26 |  |  | 16.8 |  |
| COMPOS27 |  |  | 21.1 |  |
| COMPOS28 |  |  |  | Pos |
| COMPOS29 |  |  | 16.7 |  |
| COMPOS3 |  |  | 19.1 |  |
| COMPOS30 |  |  | 20.1 |  |
| COMPOS31 |  |  | 22.1 |  |
| COMPOS32 |  |  | 21.3 |  |
| COMPOS33 |  |  | 23.7 |  |
| COMPOS34 |  |  | 16 |  |
| COMPOS35 |  |  | 24.3 |  |
| COMPOS37 |  |  | 33.8 |  |
| COMPOS38 |  |  |  | Pos |
| COMPOS39 |  |  | 21.7 |  |
| COMPOS40 |  |  |  | Pos |
| COMPOS41 |  |  | 18.2 |  |
| COMPOS42 |  |  | 23 |  |
| COMPOS43 |  |  |  | Pos |
| COMPOS44 |  |  | 23.9 |  |
| COMPOS45 |  |  | 20.1 |  |
| COMPOS46 |  |  |  | Pos |
| COMPOS47 |  |  | 17.7 |  |
| COMPOS49 |  |  | 17.2 |  |
| COMPOS51 |  |  | 28.3 |  |
| COMPOS52 |  |  | 15.5 |  |
| COMPOS6 |  |  | 16.7 |  |
| COMPOS7 |  |  | 14 |  |
| COMPOS8 |  |  | 14.9 |  |
| COMPOS9 |  |  | 19.6 |  |
| HOSPOS1 |  |  |  | Pos |
| HOSPOS10 | 37.0 | 37.0 |  |  |
| HOSPOS11 |  |  | 34.2 |  |
| HOSPOS12 | 28.8 | 29.5 |  |  |
| HOSPOS13 | 33.8 | 35.2 |  |  |
| HOSPOS14 | 23.2 | 23.1 |  |  |
| HOSPOS15 | 37 | 34.6 |  |  |
| HOSPOS16 |  |  | 27.3 |  |
| HOSPOS17 | 18.1 | 18.2 |  |  |
| HOSPOS18 | 27.5 | 27.8 |  |  |
| HOSPOS19 | 31.3 | 32.1 |  |  |
| HOSPOS2 | 24.4 | 29.1 |  |  |
| HOSPOS20 | 34.2 | 35.3 |  |  |
| HOSPOS21 |  |  |  | Pos |
| HOSPOS22 | 34.2 | 35.3 |  |  |
| HOSPOS23 |  |  |  | Pos |
| HOSPOS24 |  |  |  | Pos |
| HOSPOS25 | 27.5 | 28.1 |  |  |
| HOSPOS26 | 28.3 | 28.3 |  |  |
| HOSPOS27 | 27.3 | 27.5 |  |  |
| HOSPOS3 |  |  | 34.8 |  |
| HOSPOS30 | 15.5 | 15.7 |  |  |
| HOSPOS31 |  |  |  | Pos |
| HOSPOS32 |  |  |  | Pos |
| HOSPOS33 |  |  |  | Pos |
| HOSPOS34 | 28.6 | 29.6 |  |  |
| HOSPOS35 | 20.3 | 21.0 |  |  |
| HOSPOS36 | 25.3 | 25.8 |  |  |
| HOSPOS37 |  |  |  | Pos |
| HOSPOS38 |  |  |  | Pos |
| HOSPOS39 |  |  |  | Pos |
| HOSPOS4 |  |  | 27.4 |  |
| HOSPOS40 | 30.6 | 30.5 |  |  |
| HOSPOS41 | 33.3 | 32.9 |  |  |
| HOSPOS42 |  | 33.3 |  |  |
| HOSPOS44 |  |  |  | Pos |
| HOSPOS45 | 21.3 | 21.9 |  |  |
| HOSPOS46 |  |  |  | Pos |
| HOSPOS47 | 33.8 | 34.2 |  |  |
| HOSPOS48 |  |  |  | Pos |
| HOSPOS49 | 29.2 | 29.7 |  |  |
| HOSPOS5 | 37 | 34.6 |  |  |
| HOSPOS50 |  |  |  | Pos |
| HOSPOS53 |  |  |  | Pos |
| HOSPOS6 |  |  | 27.3 |  |
| HOSPOS7 | 18.9 | 19.1 |  |  |
| HOSPOS8 | 20.0 | 20.4 |  |  |
| HOSPOS9 | 33.4 | 33.8 |  |  |

**
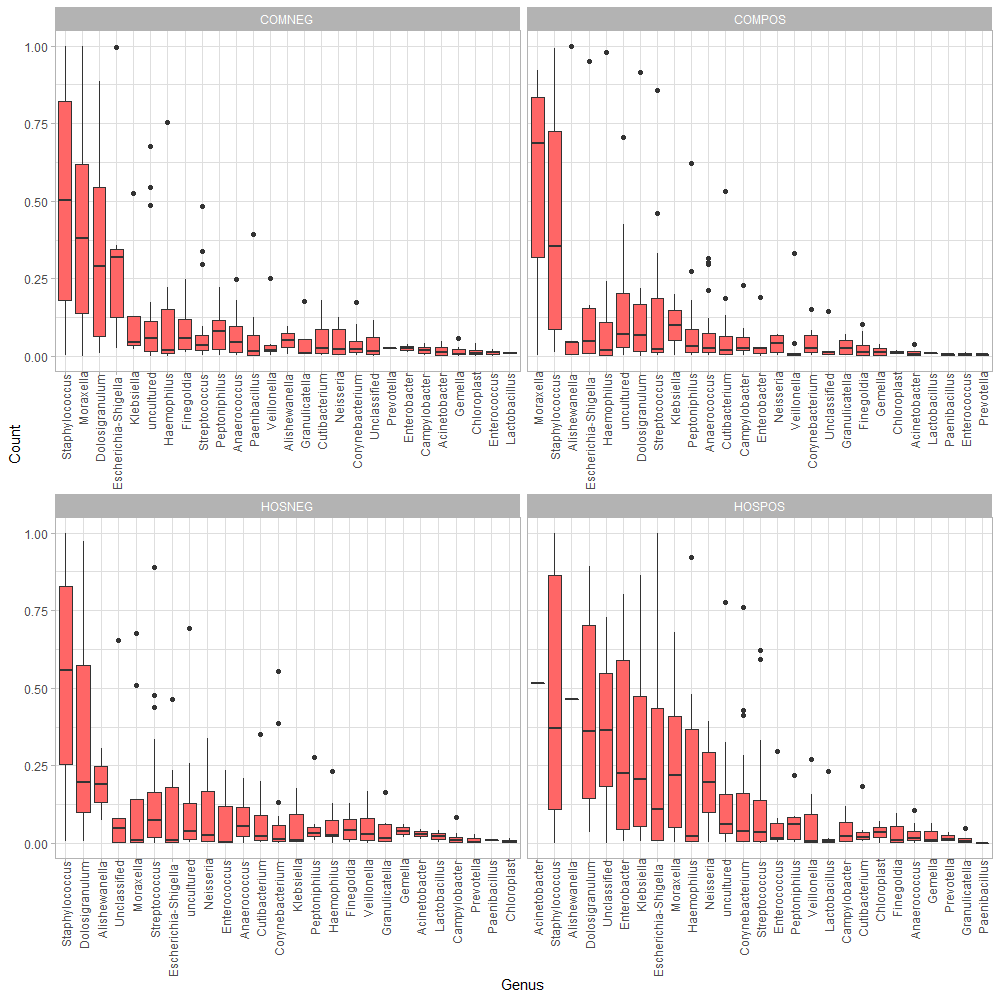
**

**S2 Fig. Genus-level relative abundance.** Side-by-side boxplots of relative abundance at the genus-level among our four study groups. Only taxa with an overall samples prevalence greater than 5% are shown. Taxa are ordered from highest to lowest mean relative abundance.

**
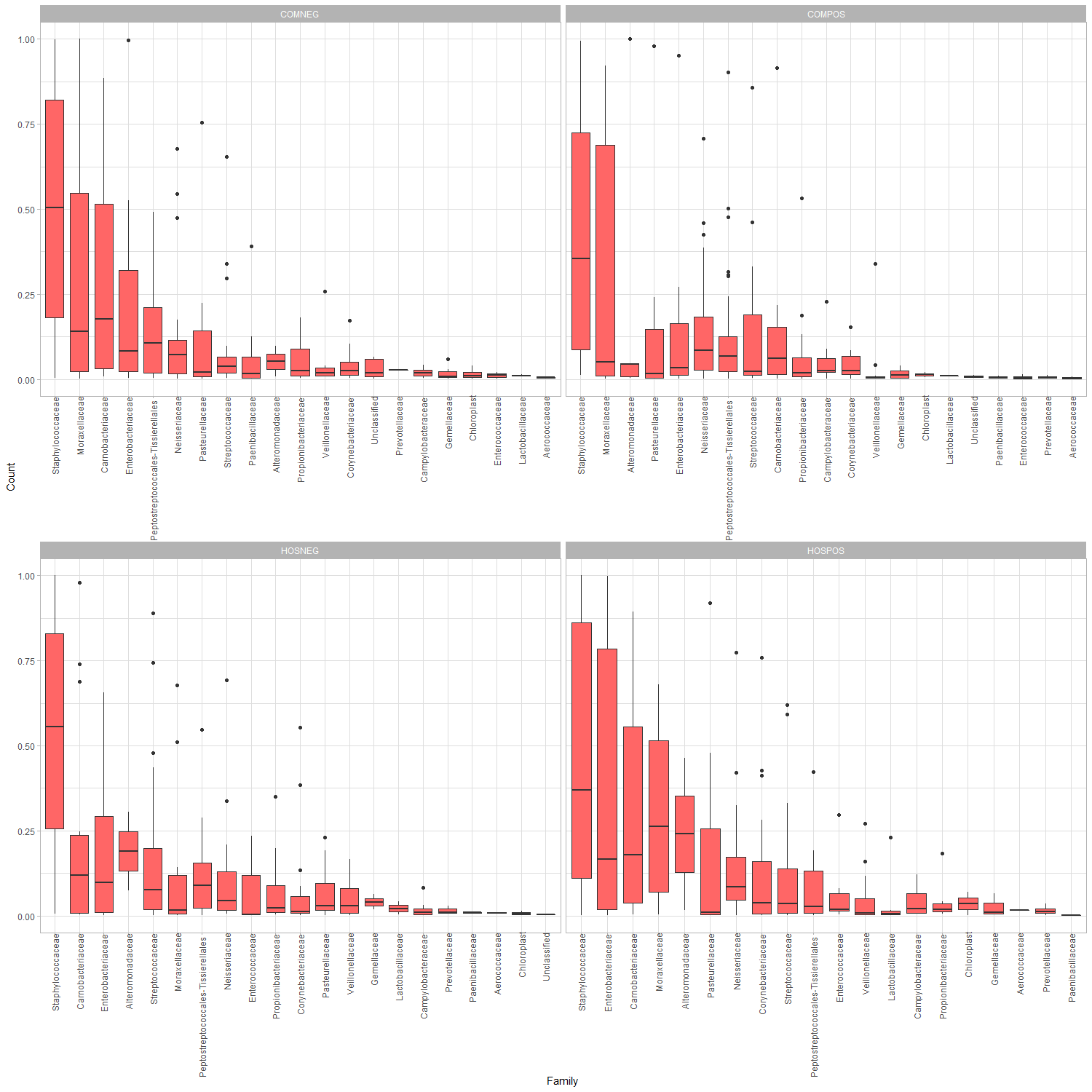
**

**S3 Fig. Family-level relative abundance.** Side-by-side boxplots of relative abundance at the family-level among our four study groups. Only taxa with an overall samples prevalence greater than 5% are shown. Taxa are ordered from highest to lowest mean relative abundance.
